# AVPR1A distribution in the whole C57BL/6J mouse neonate

**DOI:** 10.1038/s41598-020-71392-1

**Published:** 2020-09-03

**Authors:** Katherine R. Day, Alexis Coleman, Maria A. Greenwood, Elizabeth A. D. Hammock

**Affiliations:** Department of Psychology and Program in Neuroscience, The Florida State University, 1107 West Call Street, Tallahassee, FL 32306 USA

**Keywords:** Developmental biology, Neuroscience

## Abstract

The neuropeptide arginine vasopressin (AVP) plays significant roles in maintaining homeostasis and regulating social behavior. In vaginally delivered neonates, a surge of AVP is released into the bloodstream at levels exceeding release during life-threatening conditions such as hemorrhagic shock. It is currently unknown where the potential sites of action are in the neonate for these robust levels of circulating AVP at birth. The purpose of this study is to identify the location of AVP receptor 1a (AVPR1A) sites as potential peripheral targets of AVP in the neonatal mouse. RT-qPCR analysis of a sampling of tissues from the head demonstrated the presence of *Avpr1a* mRNA, suggesting local peripheral translation. Using competitive autoradiography in wildtype (WT) and AVPR1A knockout (KO) postnatal day 0 (P0) male and female mice on a C57BL/6J background, specific AVPR1A ligand binding was observed in the neonatal mouse periphery in sensory tissues of the head (eyes, ears, various oronasal regions), bone, spinal cord, adrenal cortex, and the uro-anogenital region in the neonatal AVPR1A WT mouse, as it was significantly reduced or absent in the control samples (AVPR1A KO and competition). AVPR1A throughout the neonatal periphery suggest roles for AVP in modulating peripheral physiology and development of the neonate.

## Introduction

The transition from aquatic to terrestrial life during mammalian birth represents an exceptionally vulnerable time in development^1^. The neuropeptide arginine vasopressin (AVP, also known as antidiuretic hormone) may play a role in the preparation for this perinatal transition. While AVP is well known for its role in maintaining homeostasis and modulating social behavior in adult mammals, during vaginal delivery human infants experience a massive surge of circulating AVP^2,3^ that will surpass known increases of AVP in response to other stressors in life^4^. AVP is hypothesized to allow a neonate to successfully transition from the secure support of the placenta and maternal oxygen supply into the external world. AVP has been associated with birth-related processes in the infant such as fluid control via delaying voiding^5^, reduction of lung fluid^6^, and vasoconstriction and direction of blood to vital organs^7^. During the obligatory prolonged hypoxic and hypercapnic asphyxia that is experienced by a neonate during vaginal delivery, the surge of AVP may offer protection to tissues that are sensitive to oxygen deprivation^8,9^ and promote analgesia in the neonate^10^.

AVP can act upon AVP receptor 1a (AVPR1A), AVP receptor 1b (AVPR1B), AVP receptor 2 (AVPR2), and oxytocin receptor (OXTR)^11^. AVPR1A is located throughout the central and peripheral nervous systems of neonatal and adult mammals while AVPR1B is localized in corticotrophs of the anterior pituitary and in the hippocampus. AVPR2 is found predominantly in the kidney. AVPR1A has been identified as a main receptor responsible for numerous social behaviors such as pair bonding in monogamous species^12^, social memory^13^, and modulation of odor learning in infant mice^14^. Additionally, AVPR1A has been linked to neonatal oxytocin (OXT) and AVP induced analgesia^15^. We have previously mapped OXTR in the periphery of the neonatal mouse^16,17^ improving our mechanistic hypotheses of neonatal neuropeptide function. To better understand the organs and tissues throughout the body most likely to respond directly to the surge of circulating AVP during vaginal birth, in this study we identified prominent locations of AVPR1A binding sites throughout the periphery of the neonatal mouse.

## Materials and methods

### Animals

*Avpr1a* mice^18^, fully backcrossed to C57BL/6J^19^, were bred in house. All breeding and experimental protocols were performed with the approval of Institutional Animal Care and Use Committee of Florida State University (protocols 1722 and 1746) in accordance with state and federal guidelines (Guide for the Care and Use of Laboratory Animals of the National Institutes of Health). Breeder pairs of heterozygous *Avpr1a* mice were housed in a temperature and humidity-controlled environment, on a 12 h light/dark cycle with food and water available ad libitum in open air wire-top caging. Pregnancies were not timed, but breeders were checked daily, and the first appearance of a litter was defined as postnatal day 0 (P0). More than 7 litters were collected from multiple breeder pairs, with multiple litters contributing to the sample size for each genotype to reduce litter effects. On P0, parents were removed from the cage and the litter was euthanized with either decapitation for RT-qPCR or prolonged CO_2_ exposure for autoradiography. Weight and tail samples were collected prior to freezing the whole body tissue in liquid nitrogen or isopentane on dry ice. Samples were stored at − 80 °C until microdissection or cryosectioning.

### Genotype and sex determination

Genetic sex and *Avpr1a* genotype of each member of the litter were determined by PCR on tail samples. To determine genetic sex and *Avpr1a genotypes*, previously established methods^19,20^ were used with the primers listed in Supplemental Table 1.

### RT-qPCR

Whole head samples were coronally cryosectioned at 60 μm until the region of interest (ROI) was visible and microdissected. Tissue samples collected for mRNA analysis included the mouth, eye, cortex, hippocampus, hypothalamus, and trigeminal ganglia. ROIs were removed via microdissection with a scalpel and homogenized in Trizol (Invitrogen 15596-026, CA, USA). RNA was isolated with chloroform extraction and alcohol precipitation. RNA quantity was measured using a Nanodrop Spectrophotometer ND-1000 (Thermo Fisher Scientific, USA) and RNA integrity was evaluated by agarose electrophoresis. cDNA was synthesized from DNase-treated RNA with reverse transcriptase enzyme (Applied Biosystems 4319983, CA, USA) and primer (Applied Biosystems 4319979, CA, USA). SYBR (Bio Rad 170-8882, CA, USA) was used along with the primers listed in Supplemental Table 2 to detect *Avpr1a*. Primers targeted the *Avpr1a* transcript NM_016847.2 exon 1 at 1,241–1,330 bp and exon 2 at 1548–1619 bp. AVPR1A KO mice only have genomic exon 2, as exon 1 is removed^18^.

### Receptor autoradiography

Whole body samples were cryosectioned in the sagittal plane in 8 series of 20 μm. Tissue was mounted on SuperFrost Plus slides (VWR) and stored at − 80 °C. Standard methods^19^ were used to perform receptor autoradiography with 0.05 nM ^125^I labeled linear AVP receptor ligand (AVPR1A antagonist NEX310; NEN/Perkin-Elmer, Waltham, MA^21,22^). To competitively assess nonspecific binding in adjacent sets of slides from WT and AVPR1A KO mice, unlabeled AVP peptide (V9879, Sigma Aldrich) was added to half of the tracer buffer to yield two concentrations of unlabeled AVP competition (0 nM and 1000 nM). Autoradiographic films (Kodak Biomax MR film, Carestream Health, Inc., Rochester, NY, USA) were exposed to slides and ^14^C autoradiographic standards (ARC-0146; American Radiolabeled Chemicals, St. Louis, MO, USA) using previously established methods^17^ for one 3-day period and one 10-day period before developing (Mini-Medical/90 X-ray film processor, AFP Imaging, New York).

### Image analysis

After autoradiography, slides were processed with cresyl violet (CV) for unbiased ROI measurements and anatomical reference. A flatbed scanner (EPSON, Epson Perfection V600 Photo) was used to scan slides and film at 1,200 dpi. ROIs were identified on CV stained slides and measurements were collected from corresponding film images. Image inversion was the only image adjustment, so higher numbers represented more dense binding. The TurboReg^23^ plugin was used to create composite images using the rigid-body alignment algorithm. For pseudocolor composites, the autoradiography images were adjusted for brightness to minimize the appearance of the film background. ImageJ (NIH, Bethesda, MD) was used for quantification. For whisker pad quantification, the δ whisker follicle was identified^24^ and measured by using the straight line selection tool to create a line across the diameter of the follicle and the values along the line were averaged. Three distinct morphologies of whisker pad were identified and quantified separately; level 1 (0–160 μm) at the epidermis, level 2 (160–480 μm) in the follicular intrinsic muscle, and level 3 (480–640 μm), at the base of the whisker follicle. For quantification of all other ROIs the brush-selection tool was used to select the entire ROI from three consecutive sections within each animal, which were averaged. To generate local background-corrected densitometry values, local values from non-tissue background were obtained from the slide containing the ROI, measured in a 20 × 20 pixel region, then subtracted from the ROI values. For any ROI where film background showed higher density than the ROI, the ROI binding value was set to 0. This process was repeated using ^14^C autoradiographic standard on the same film^25^ to generate a linear range of standardized values. Ligand binding values (μCi/gram) were calculated by interpolation “interp1,” MatLab 8.1.0 (TheMathworks, Natick, MA, USA) to the linear range of the ^14^C autoradiographic standard on the same film^25^.

### RT-qPCR statistics

Our sample size included 6 WT (3 male) and 2 AVPR1A KO (1 male) mice. Omnibus ANOVA was used to determine if there were ROI differences in the GAPDH cycle time (Ct) values. A non-parametric Wilcoxon signed-rank test was used for each ROI to determine if WT relative expression was above the KO baseline value.

### Autoradiography statistics

A 2 (genotype) × 2 (competition dose) mixed-design ANOVA with competition dose as a within-subjects factor and Bonferroni corrections for multiple post-hoc comparisons in GraphPad Prism version 8.00 for Windows (GraphPad Software, La Jolla, CA) was used to determine if ROIs had specific AVPR1A binding. Our sample sizes included 5 WT (3 male) and 5 AVPR1A KO (2 male) mice. Sex was not included as an independent variable in our statistical analysis, as this study is underpowered to observe sex differences. Our a priori threshold for determining specific AVPR1A ligand binding was the absence of specific signal in the AVPR1A KO as well as displacement of the unlabeled competitive ligand in AVPR1A WT neonates was our a priori threshold, as reflected in statistically significant genotype × dose interactions. On tissues that did not show a significant interaction term, a secondary exploratory analysis was performed on WT data alone with a paired t-test to evaluate competition.

## Results

### RT-qPCR

Primer efficiency was determined by comparison of cDNA dilutions and Ct to generate standard curves which were parallel for all primers. *Gapdh* r^2^ was 0.991 with a primer efficiency of 72.39%. *Avpr1a exon 1* r^2^ was 0.966 with a primer efficiency of 88.97%. *Avpr1a exon 2* r^2^ was 0.946 with a primer efficiency of 89.64%. The Ct values from the *Gapdh* primer set were analyzed to confirm that there was no bias in genotype or tissue source. First, *Gapdh* Ct values were normally distributed according to a Shapiro–Wilk (SW) test (SW-test = 0.973, *p* = 0.341). Second, there were no significant main effects for genotype (F_1,5_ = 0.425; *p* = 0.519) or region (F_1,5_ = 1.553; *p* = 0.198) or interactions between genotype and region (F_1,5_ = 0.297; *p* = 0.911) for *Gapdh*, indicating its utility as a reference gene for these samples.

**Figure 1:**
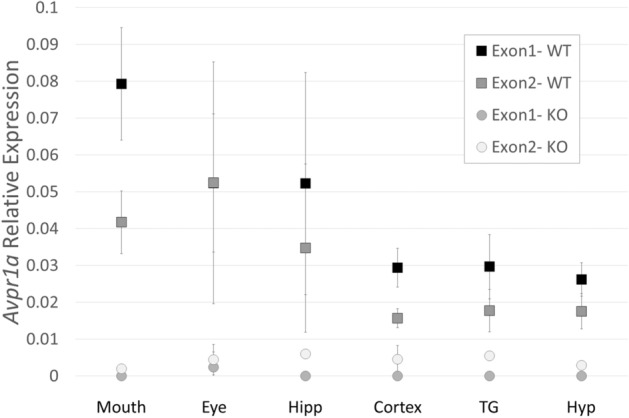
*Avpr1a* mRNA is present in measured areas of the neonatal (P0) mouse head detected by RT-qPCR. *Avpr1a* expression relative to *Gapdh* (2^−dCt^ method) was evident in all six regions of interest from WT mice (n = 6) [mouth, eye, hippocampus (Hipp), cortex, trigeminal ganglion (TG) and hypothalamus (Hyp)] when compared to *Avpr1a* KO mice (n = 2). Data are mean ± SEM.

The relative expression (compared to *Gapdh*; 2^−dCt^ method) of the *Avpr1a exon 1* and the *Avpr1a exon 2* primers had a non-normal distribution according to the SW test (*Avpr1a Exon 1* SW-test = 0.758, *p* < 0.001; *Avpr1a Exon 2* SW-test = 0.530, *p* < 0.001). Data would not yield to transformation, and the expression from the two primer sets were highly correlated (Pearson’s r = 0.849, *p* < 0.001) so a one sample, one sided Wilcoxon signed-rank test was performed on the combined average expression of *Avpr1a Exon 1* and *Avpr1a Exon 2* for the WT data from each ROI to determine if WT expression was higher than the KO baseline (set to the average amplification of 0.002). As expected, the *Avpr1a* WT mice showed significantly more expression than the *Avpr1a* KO baseline with each ROI yielding the same result (Wilcoxon (d.f. 5) = 21.0; *p* = 0.016), indicating that the expression observed was indicative of true expression and not noise. A combined summary of relative expression of the two primers of all ROIs from both sexes can be found in Fig. 1. As expected, the AVPR1A KO samples did not show amplification above background of the *Avpr1a exon 1* primer set but did show modest amplification with the *Avpr1a exon 2* primer set, which is still intact in the AVPR1A KO genome. To control for the possibility of gDNA contamination, both primer sets were run on WT and KO RNA samples that had not undergone cDNA synthesis. Neither primer set resulted in qPCR amplification of this non-cDNA material (not shown).

Control tissues from known regions of *Avpr1a* expression in the brain (hippocampus, cortex, and hypothalamus) performed as expected with expression in the WT compared to KO samples with both primer sets. Tissues dissected from peripheral ROI of the head including the mouth, eye, and trigeminal ganglion all demonstrated *Avpr1a* mRNA expression in the WT neonate compared to KO tissue. Determination of expression in these peripheral regions encouraged a pursuit of the autoradiography approach.

### Autoradiography

**Figure 2:**
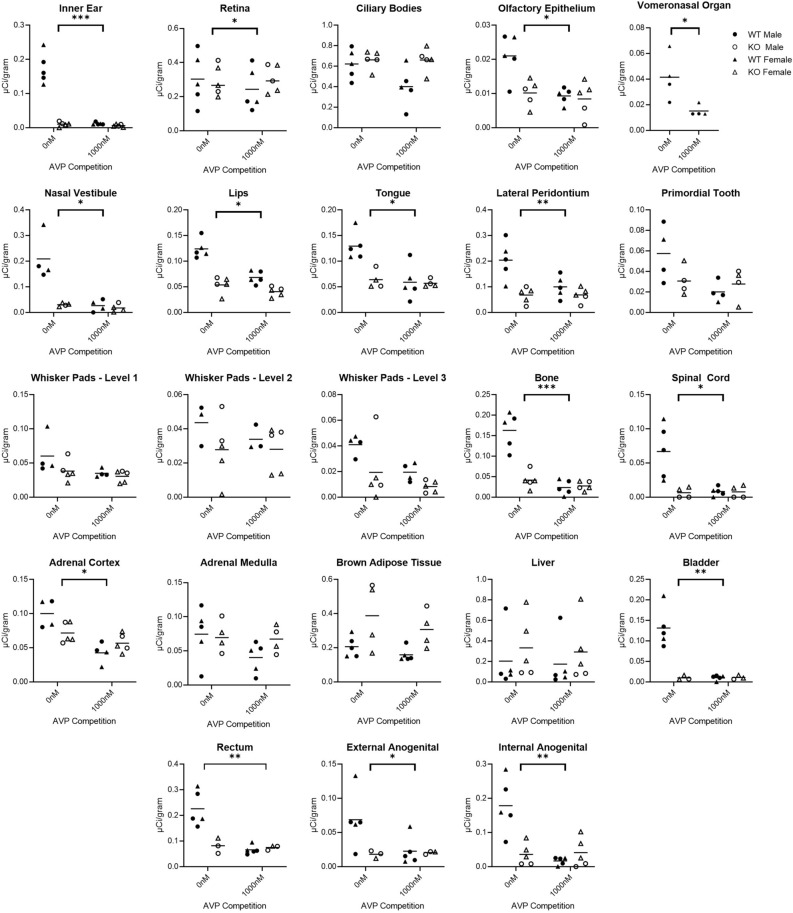
Differences between wild-type (WT) and knockout (KO) genotypes are evident in the 0 nM AVP competition condition, with displacement of binding in the WT 1000 nM AVP competition. WT and KO group means, and individual subject data are plotted [WT (filled symbols), AVPR1A KO (open symbols), males (circles), females (triangles)]. The y-axis of each graph has been fit to the scale of the data. Significant genotype × dose interaction terms are indicated, except for VNO, where only an effect of dose could be tested in WT. * indicates *p* ≤ 0.05, ** indicates *p* ≤ 0.01, *** indicates *p* ≤ 0.001.

**Figure 3:**
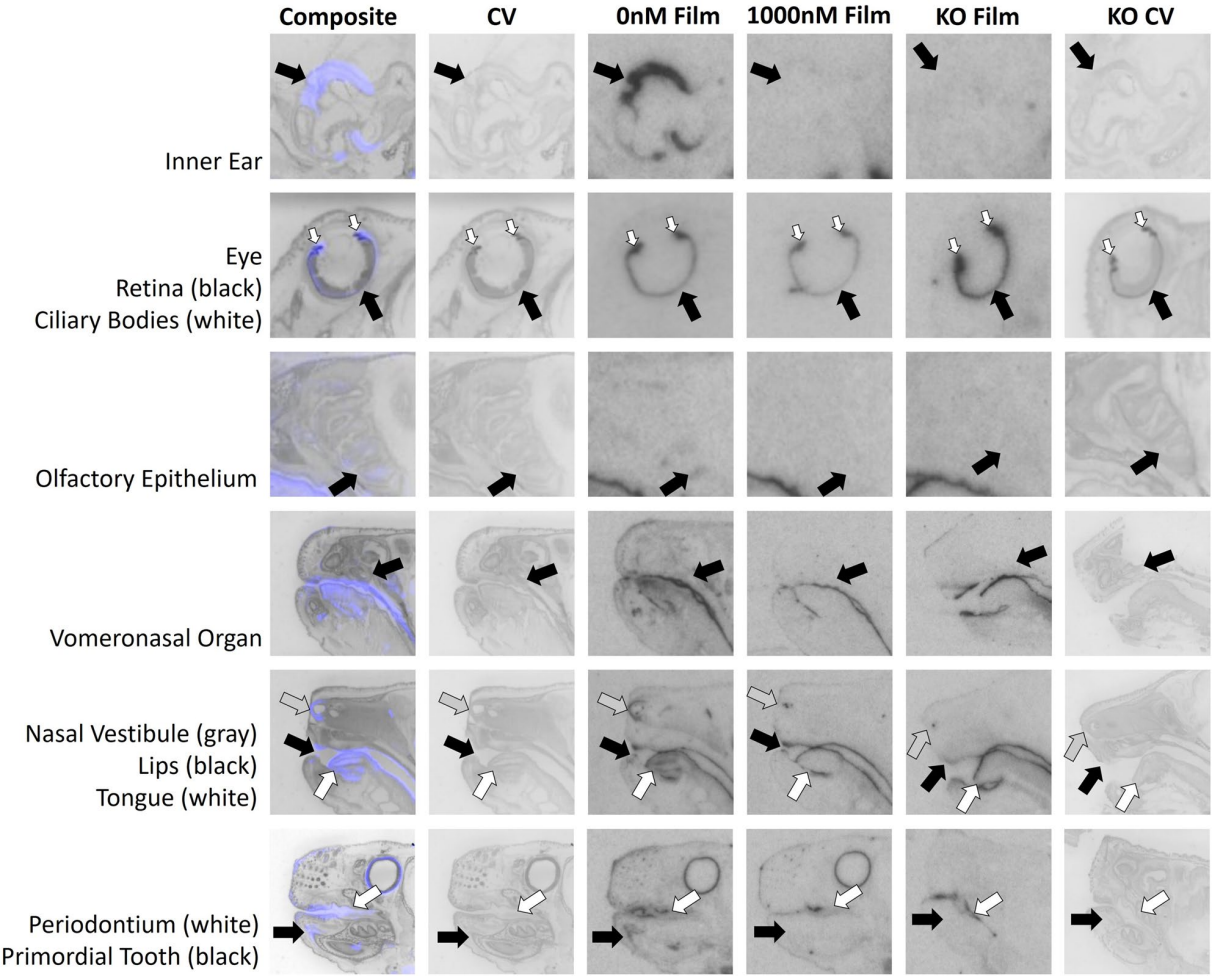
AVPR1A ligand binding present in tissues of the mouse neonatal head at P0. Arrows indicate region of interest. Composite = AVPR1A ligand binding and anatomy in a WT neonate (AVPR1A pseudo-colored in purple, cresyl violet counterstain in gray); CV = post-processed anatomical cresyl violet counterstain; 0 nM Film = AVPR1A ligand binding with no AVP competition in a WT neonate; 1000 nM Film = AVPR1A ligand binding in the 1000 nM AVP competition in a WT neonate; KO Film = AVPR1A ligand binding in AVPR1A KO mice; KO CV = post-processed anatomical cresyl violet stain of the AVPR1A KO neonate.

**Figure 4:**
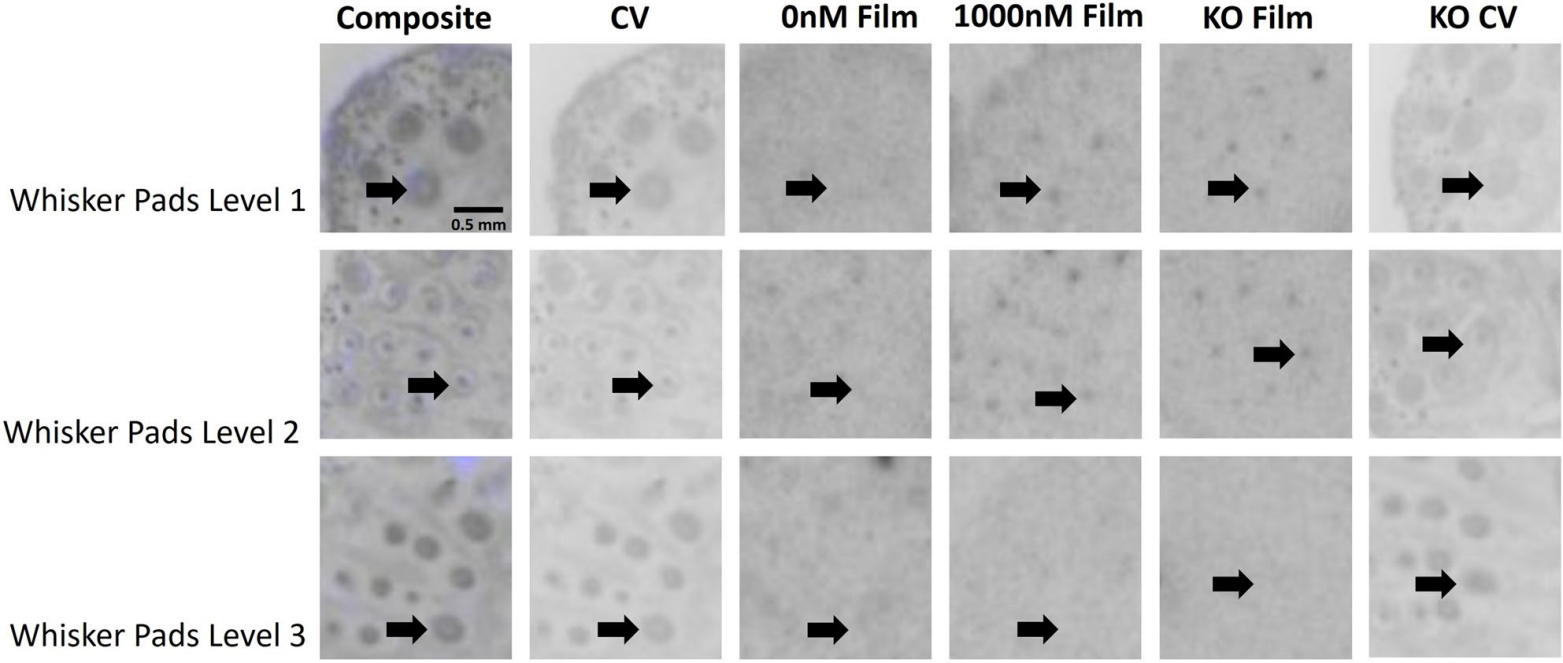
Evaluation of AVPR1A ligand binding in the whisker pad of the neonatal mouse at P0. Arrows indicate region of interest. Composite = AVPR1A ligand binding and anatomy in a WT neonate (AVPR1A pseudo-colored in purple, cresyl violet counterstain in gray); CV = post-processed anatomical cresyl violet counterstain; 0 nM Film = AVPR1A ligand binding with no AVP competition in a WT neonate; 1000 nM Film = AVPR1A ligand binding in the 1000 nM AVP competition in a WT neonate; KO Film = AVPR1A ligand binding in AVPR1A KO mice; KO CV = post-processed anatomical cresyl violet stain of the AVPR1A KO neonate.

**Figure 5:**
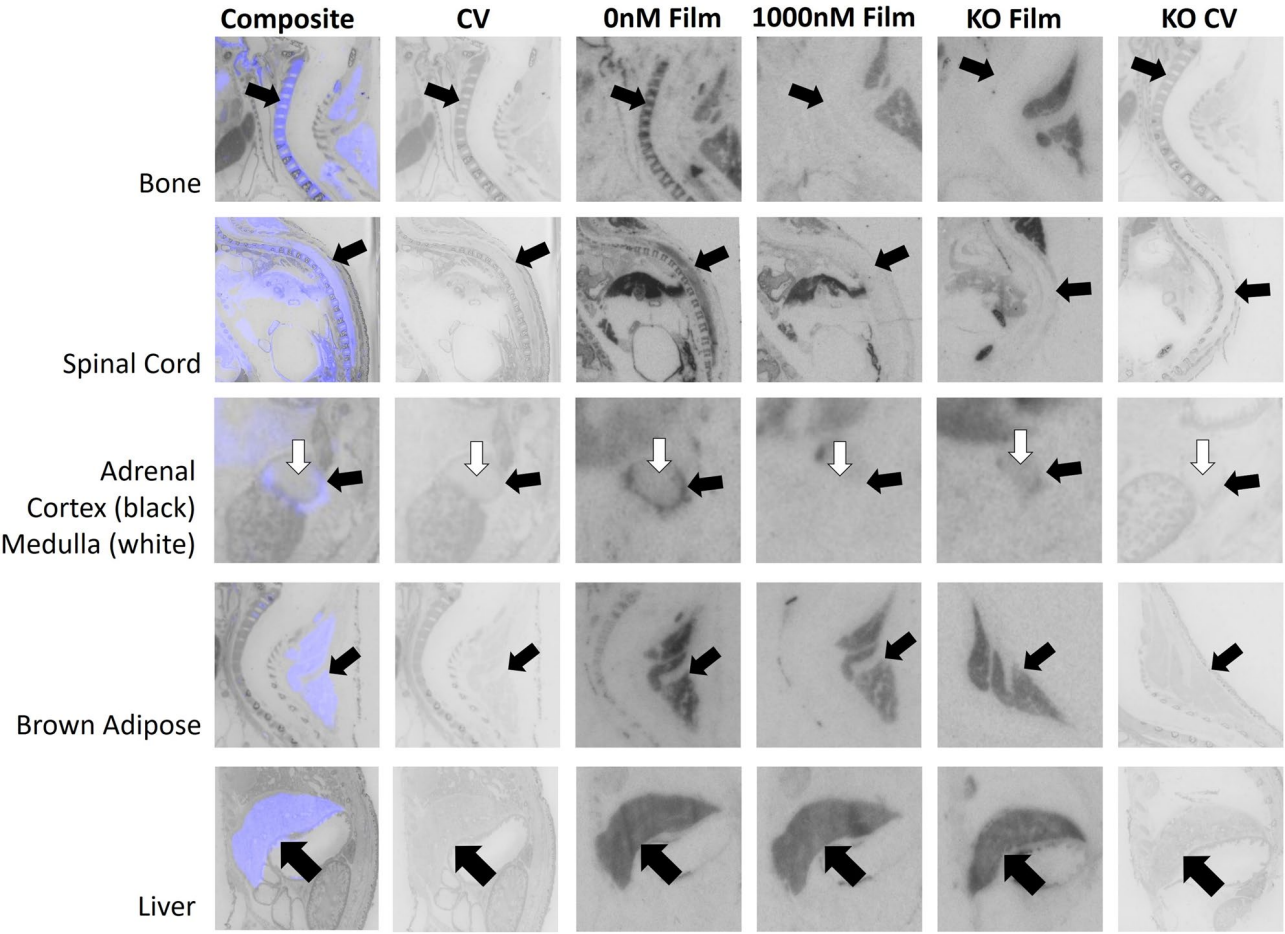
AVPR1A ligand binding is evident in the periphery of the neonatal mouse at P0. The bone, spinal cord, and adrenal cortex all showed evidence of specific activity, while the brown adipose tissue and liver did not. Arrows indicate region of interest. Composite = AVPR1A ligand binding and anatomy in a WT neonate (AVPR1A pseudo-colored in purple, cresyl violet counterstain in gray); CV = post-processed anatomical cresyl violet counterstain; 0 nM Film = AVPR1A ligand binding with no AVP competition in a WT neonate; 1000 nM Film = AVPR1A ligand binding in the 1000 nM AVP competition in a WT neonate; KO Film = AVPR1A ligand binding in AVPR1A KO mice; KO CV = post-processed anatomical cresyl violet stain of the AVPR1A KO neonate.

**Figure 6:**
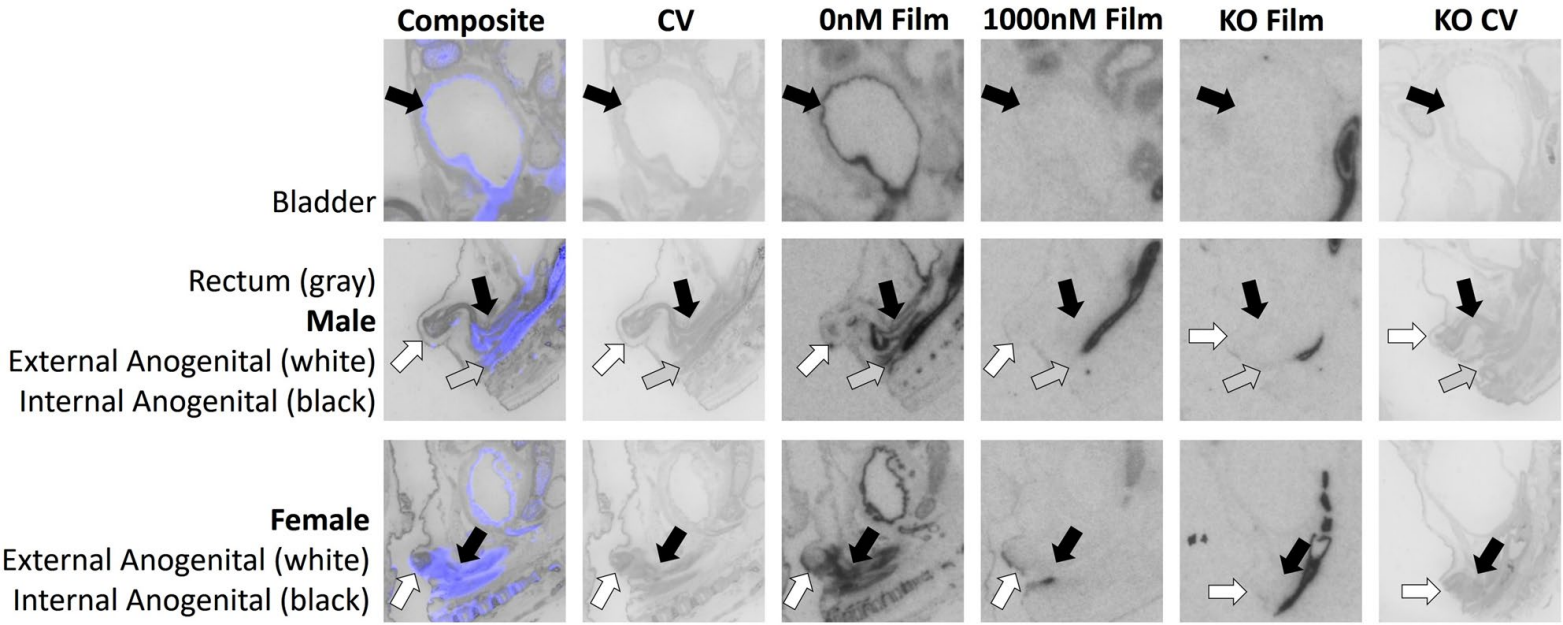
AVPR1A ligand binding is evident in the genitourinary system and rectum of the neonatal mouse at P0. Arrows indicate region of interest. Composite = AVPR1A ligand binding and anatomy in a WT neonate (AVPR1A pseudo-colored in purple, cresyl violet counterstain in gray); CV = post-processed anatomical cresyl violet counterstain; 0 nM Film = AVPR1A ligand binding with no AVP competition in a WT neonate; 1000 nM Film = AVPR1A ligand binding in the 1000 nM AVP competition in a WT neonate; KO Film = AVPR1A ligand binding in AVPR1A KO mice; KO CV = post-processed anatomical cresyl violet stain of the AVPR1A KO neonate.

We quantified receptor binding in the inner ear, retina, ciliary bodies, olfactory epithelium, tissues surrounding the nasal vestibule, lips, tongue, periodontium, primordial tooth, whisker pads, vertebral bone, spinal cord, adrenal medulla, adrenal cortex, brown adipose tissue, liver, bladder, rectum, external anogenital area, and internal anogenital area. Quantitative densitometry for each ROI can be found in Fig. 2. Males and females were analyzed together; however, they were plotted individually in Fig. 2. Statistical results for two-way ANOVA for each ROI are presented in Table 1, using Bonferroni corrected *p*-values. Exploratory analyses using paired t-tests are presented in Table 2. Representative images are presented in Figs. 3, 4, 5 and 6.

**Table 1:** Two-way ANOVA with Bonferroni corrected *p*-values.

Region of interest	Independent variables	F-value (df)	*p*-value
Inner ear	Genotype	F(1, 8) = 61.80	< .001
	Dose	F(1, 8) = 70.50	< .001
	Genotype × dose	F(1, 8) = 63.60	< .001
Retina	Genotype	F(1, 8) = 0.13	0.727
	Dose	F(1, 8) = 4.19	0.075
	Genotype × dose	F(1, 8) = 7.11	0.029
Ciliary bodies	Genotype	F(1, 8) = 5.38	0.049
	Dose	F(1, 8) = 3.42	0.102
	Genotype × dose	F(1, 8) = 3.29	0.107
Olfactory epithelium	Genotype	F(1, 8) = 6.87	0.031
	Dose	F(1, 8) = 11.00	0.011
	Genotype × dose	F(1, 8) = 6.02	0.040
Nasal vestibule	Genotype	F(1, 6) = 17.50	0.006
	Dose	F(1, 6) = 15.60	0.008
	Genotype × dose	F(1, 6) = 11.80	0.014
Lips	Genotype	F(1, 8) = 77.50	0.001
	Dose	F(1, 8) = 21.70	0.002
	Genotype × dose	F(1, 8) = 8.19	0.021
Tongue	Genotype	F(1, 7) = 6.14	0.042
	Dose	F(1, 7) = 15.70	0.005
	Genotype × dose	F(1, 7) = 10.50	0.014
Lateral periodontium	Genotype	F(1, 8) = 9.68	0.014
	Dose	F(1, 8) = 14.70	0.005
	Genotype × dose	F(1, 8) = 15.10	0.005
Primordial tooth	Genotype	F(1, 6) = 1.28	0.302
	Dose	F(1, 6) = 4.50	0.078
	Genotype × dose	F(1, 6) = 3.29	0.120
Whisker pads level 1	Genotype	F(1, 7) = 1.92	0.208
	Dose	F(1, 7) = 7.83	0.027
	Genotype × dose	F(1, 7) = 2.17	0.184
Whisker pads level 2	Genotype	F(1, 6) = 1.23	0.309
	Dose	F(1, 6) = 1.32	0.294
	Genotype × dose	F(1, 6) = 1.44	0.275
Whisker pads level 3	Genotype	F(1, 7) = 4.93	0.062
	Dose	F(1, 7) = 7.03	0.033
	Genotype × dose	F(1, 7) = 0.71	0.427
C5 vertebrae	Genotype	F(1, 8) = 26.20	0.001
	Dose	F(1, 8) = 38.40	0.001
	Genotype × dose	F(1, 8) = 25.5	0.001
Spinal cord	Genotype	F(1, 7) = 8.01	0.025
	Dose	F(1, 7) = 8.02	0.025
	Genotype × dose	F(1, 7) = 8.69	0.021
Adrenal medulla	Genotype	F(1, 7) = 0.52	0.494
	Dose	F(1, 7) = 4.46	0.073
	Genotype × dose	F(1, 7) = 2.60	0.151
Adrenal cortex	Genotype	F(1, 7) = 1.16	0.317
	Dose	F(1, 7) = 18.10	0.004
	Genotype × dose	F(1, 7) = 6.27	0.041
Brown adipose tissue	Genotype	F(1, 7) = 5.64	0.049
	Dose	F(1, 7) = 5.28	0.055
	Genotype × dose	F(1, 7) = 0.35	0.573
Liver	Genotype	F(1, 8) = 0.48	0.510
	Dose	F(1, 8) = 3.29	0.107
	Genotype × dose	F(1, 8) = 0.08	0.784
Bladder	Genotype	F(1, 6) = 16.40	0.007
	Dose	F(1, 6) = 19.30	0.005
	Genotype × dose	F(1, 6) = 19.90	0.004
Rectum	Genotype	F(1, 6) = 6.84	0.040
	Dose	F(1, 6) = 22.10	0.003
	Genotype × dose	F(1, 6) = 18.50	0.005
External anogenital	Genotype	F(1, 6) = 2.11	0.197
	Dose	F(1, 6) = 9.14	0.023
	Genotype × dose	F(1, 6) = 11.30	0.015
Internal anogenital	Genotype	F(1, 8) = 5.31	0.050
	Dose	F(1, 8) = 21.40	0.002
	Genotype × dose	F(1, 8) = 24.40	0.001

Regions with genotype × dose interaction of *p* < 0.05 were considered to have specific AVPR1A binding.

**Table 2:** Student’s *t*-test.

Region of interest	Independent variable	t- statistic (df)	*p*-value
Adrenal medulla	Dose	2.53 (4)	0.065
Brown adipose tissue	Dose	1.54 (4)	0.199
Ciliary bodies	Dose	1.87 (4)	0.134
Liver	Dose	1.84 (4)	0.139
Primordial tooth	Dose	2.12 (3)	0.124
Whisker pads level 1	Dose	2.16 (3)	0.119
Whisker pads level 2	Dose	1.76 (2)	0.220
Whisker pads level 3	Dose	3.68 (3)	0.035
VNO	Dose	3.63 (3)	0.036

Regions without significant genotype × dose interactions were queried with an exploratory Student’s *t*-test to investigate dose- dependent effects in WT only, as this is the criteria for specificity when a KO model is not available. Regions with a dose-dependent *p* < 0.05 may have significant AVPR1A binding in WT tissue.

A significant genotype × dose interaction was pre-defined as a criterion marker for robust specific AVPR1A ligand binding activity for a given ROI, as assessed in the mixed-effects ANOVA. Of the areas that we measured, the areas in the neonatal mouse with a significant genotype × competition dose at *p* < 0.05 include the ear, retina, olfactory epithelium, tissues surrounding the nasal vestibule, lips, tongue, lateral periodontium, bone at C5 vertebrae, spinal cord, adrenal cortex, bladder, rectum, external anogenital area, and internal anogenital area (Fig. 2).

With effective competition in areas with significant binding, it is possible to have a main effect of competition dose, with the 0 nM competition group showing significantly more binding than the 1000 nM competition group (Fig. 2 and Table 1). Areas with a main effect of competition included inner ear, olfactory epithelium, tissues surrounding the nasal vestibule, lips, tongue, periodontium, retina, whisker pads at levels 1 and 3, bone at C5 vertebrae, spinal cord, adrenal cortex, bladder, rectum, external anogenital area, and internal anogenital area. Areas with a significant main effect showed significantly more binding in the 0 nM competition group than the 1000 nM competition group, as expected. No areas showed a main effect of competition with the 0 nM competition group having lower ligand binding than the 1000 nM competition group.

It was also possible for some areas to show significant genotype main effects, with WT showing more ligand binding than KO, as expected (Fig. 2 and Table 1). These areas include ear, olfactory epithelium, tissues surrounding the nasal vestibule, lips, tongue, periodontium, bone at C5 vertebrae, spinal cord, bladder, and rectum. The ciliary bodies and brown adipose tissue had a main effect of genotype, however the KO tissue showed higher levels of ligand binding, suggesting increased non-specific binding to fatty tissue in the KO animals (Fig. 2 and Table 1).

Of the areas we measured, several showed no statistically significant interaction term in the two-way ANOVA. This could be due to low power for detecting a significant interaction, or consistent with the absence of robust specific AVPR1A binding in those regions. Areas without a significant interaction term were put through an exploratory analysis to evaluate if competition within the WT tissue alone showed evidence of specific binding. This exploratory analysis was performed on the ciliary bodies, rostral primordial tooth, whisker pads, adrenal medulla, brown adipose tissue, and liver (Table 2). This exploratory analysis suggests that whisker pads at level 3 should be considered in future studies with a larger sample size to confirm the presence of specific AVPR1A ligand binding.

The vomeronasal organ (VNO) could not be analyzed by two-way ANOVA, so it was analyzed separately (Fig. 2, Table 2). This small area was prone to tissue damage, reducing the number of samples available for quantification. We were able to measure ligand binding in the VNO from 4 WT but only 1 KO. Therefore, we ran a t-test on the WT data with and without competition for the VNO. This indicated that there was significantly higher binding observed in 0 nM competition group than the 1000 nM competition group. The single KO data point was closer to the average for the WT with 1000 nM competition than the WT without. Combined, these data suggest that the VNO likely displays selective AVPR1A ligand binding in the neonatal mouse.

## Discussion

In this study we identify robust presence of AVPR1A in the murine neonatal periphery. Tissues found to have AVPR1A present, with implications in AVP’s role in the birth transition and development include eyes, ears, various regions of the oronasal cavity, vertebral bone, spinal cord, adrenal cortex, and the uro-anogenital region. These data are consistent with prior evidence of the presence of AVPR1A in the bone^26^ and spinal cord^27^, along with the high specificity of this [^125^I]-linear AVP ligand (AVPR1A antagonist NEX310^21,22^; NEN/Perkin-Elmer, Waltham, MA) for AVPR1A in the brain across rodent species^14,19,28^. Similar to previous autoradiography studies analyzing peripheral OXTR^17^, these data emphasize the value of analyzing tissue in WT and KO individuals, and using competitive ligand binding to better identify previously unconfirmed tissues. With both AVPR1A KO and competition via 1000 nM of unlabeled AVP, we have identified areas that exhibit nonspecific binding such as the liver, brown adipose tissue, ciliary body, primordial tooth, whisker pads (levels 1 and 2), and adrenal medulla. All of these regions may appear to exhibit ligand binding on autoradiographic films for WT samples but show statistically indistinguishable signal in the KO and with competition, suggesting nonspecific binding noise in these areas. Ongoing investigations may better assess the function of the AVPR1A in peripheral areas identified in this study and how it may be related to homeostasis/allostasis in the birth transition and in post-natal development. Although we have identified many regions in the neonatal periphery that contain AVPR1A, this list is neither exhaustive of all anatomical areas, nor powered to draw conclusions about potential sex differences at this time.

While other areas containing AVPR1A may exist, this study identified and quantified only the regions that were easily visible after 10-days of exposure to autoradiographic film. In regions such as brown adipose tissue, the retina, ciliary bodies, tissues surrounding the nasal vestibule, vertebral bone, liver, and rectum where binding was easily visible on the film after 3 days of exposure, and too intense to accurately quantify on the 10-day film, the 3-day film was quantified. For all other regions, the 10-day film was quantified as they were not yet visible on the autoradiographic film for quantification after only 3 days of exposure. Damaged, anatomically ill-defined, or faint regions were not included in the analysis presented in Figs. 2, 3, 4, 5 and 6. When possible, an area was quantified using three consecutive tissue sections, however by cutting sections of 20 μm in series of 8, any region less than 480 μm depth in the sagittal plane may only have sections available for analysis twice. For example, whisker pad Type 1 was only observed in the first 160 μm of sectioning, thus it was only able to be measured once per subject. Additionally, sampling availability may be reduced for certain tissues, such as the VNO, that are small and prone to damage during sectioning. As AVPR1A has previously been explored with autoradiography in the neonatal mouse brain^14,19,29^, the brain was not quantified in the autoradiography portion of our study.

Receptor autoradiography with selective ligands remains the technical standard for robust and specific identification of tissues expressing functional G protein coupled receptors. In comparison to antibody-based studies, the autoradiographic ligand continues to show high replicability and behaves as expected under appropriate control conditions as used in this report. While autoradiography demonstrates strengths in robust signal and high specificity, it clearly falls short when cellular-level detail is warranted. In contrast, in situ hybridization for specific mRNAs can provide this level of detail. Direct in situ hybridization methods for *Avpr1a* mRNA have been attempted in numerous expression databases including Genepaint, BGEM, and the Allen Brain Atlas^30–32^. All of these have expression profiles of mouse whole embryo, but no older than embryonic day E15.5. Transgenic reporter mice for the *Avpr1a* promoter driving EGFP expression are also available from GENSAT^33^. Data available for these mice show whole head immunohistochemistry for EGFP at E15.5. There is a robust amount of EGFP signal in the lip area. While these prior studies established evidence of *Avpr1a* mRNA expression in fetal tissue, our new evidence demonstrates robust AVPR1A protein in several areas in the full-term mouse neonate.

While we did not quantify the AVPR1A ligand binding in the neonatal mouse brain, because this has already been established, we did quantify the ligand binding in the spinal cord. AVPR1A has been previously identified in the gray matter (layers 1 and 2), and along the central canal of the spinal cord^27^ and dorsal root ganglia^34^ of the adult mouse. AVPR1A in the neonatal spinal cord may contribute to AVP’s analgesic effect in neonates^15^.

While it was apparent on film that there were several areas with AVPR1A signal in bone, we restricted our measurement to the C5 vertebral bone for consistent measurement. At birth, ossification of the spinal cord is still underway and cervical vertebrae 3–5^35^ have completed ossification centers at the time of birth. Oxytocin and AVP work together to regulate growth during ossification, and AVPR1A and OXTR have previously been identified as the receptors responsible for this in mice^36^.

It is possible that the AVPR1A signal is on bone in the inner ear, suggesting a repeated role of AVPR1A in bone formation. However, there is evidence that AVP is involved in endolymph homeostasis within the inner ear^37,38^. Aquaporin-2 (AQP2) is also involved in fluid homeostasis within the inner ear, and mRNA levels of AQP2 have been found to increase in response to elevated AVP^39^. It has been assumed that the relationship between AVP and AQP2 in the ear is through colocalization of AVPR2, due to their known colocalization and interactions in the kidney^40^. However, evidence confirming the presence of mRNA of AVPR2 within the inner ear has been conflicting^40–42^. *Avpr1a* mRNA was previously found in small amounts within the stria vascularis of the adult rat^40^, however further investigation to confirm receptors involved in fluid homeostasis of the ear is needed.

Presence of AVPR1A in the eye was expected due to the levels of mRNA observed, indicating the active transcription *Avpr1a*. While our previous study confirmed the presence of oxytocin receptor in the ciliary body of neonatal mice^17^, we observed nonspecific binding of AVPR1A of this area. However, we did observe specific AVPR1A ligand binding within the retina. AVP-immunoreactive cells have been previously identified in the retina of adult rats^43^. AVP type 1 receptors have previously been linked to dose and administration dependent effects on constriction and dilation of smooth muscle of the canine ciliary arterial strip in response to local administration of vasopressin to the eye^44^, as well as intraocular pressure and pupil size after peripherally and systemically administered vasopressin in rabbits^45^.

The olfactory epithelium and tissues surrounding the nasal vestibule exhibited specific AVPR1A ligand binding when comparing WT subjects to their KO and competition counterparts. We also observed potential AVPR1A binding in the VNO, when considering WT neonates only: the VNO was unfortunately commonly damaged and unavailable for thorough quantification in AVPR1A KO. Some social recognition and investigation behaviors in rodents depend on VNO-AVP interactions^46^. The olfactory epithelium was another area of interest, as it has previously been found to have dose dependent calcium release responses to AVP via AVPR1A^47^. While the presence of AVPR1A in the olfactory epithelium may have neuromodulatory effects on incoming olfactory information, it may also be present in Bowman’s glands for regulation of olfactory mucus. It is possible that some AVPR1A in the nasal cavity is on cartilage (e.g. tissues surrounding nasal vestibule area^48^). AVP is also involved in chondrogenesis of humans^49^ and rats^50^, although a specific vasopressin receptor has not yet been linked to chondrogenesis.

We have found specific AVPR1A radioligand binding in the tongue, lips, and lateral periodontium within the mouth. RT-qPCR revealed *Avpr1a* mRNA within the mouth. Regions of the mouth containing AVPR1A could be activated from endogenous or exogenous sources, such as parental saliva or breastmilk. Endogenously produced oxytocin and AVP have been identified in saliva of dogs^51^ and humans^52^. Oxytocin has been found in breastmilk^53,54^, offering another potential route for activation of oral sites of AVPR1A. The role of AVP in rat infant oral behavior has been linked to behaviors such as latching^55^. Increased levels of maternal AVP are associated with increased milk fat content and milk flow to offspring in goats^56^ and AVPR1A has also been identified in rat mammary plasma membrane^57^. Combined, these data suggest that AVP signaling could be available to both the mother and offspring during infant feeding.

Specific AVPR1A radioligand binding was seen in the adrenal cortex, but not the adrenal medulla. The presence of AVPR1A in the adrenal cortex has previously been reported in mice^58^, offering additional routes for AVP to activate the adrenal gland in the hypothalamic–pituitary–adrenal axis, in addition to anterior pituitary release. Intriguingly, AVPR1A is present in the adrenal medulla of rats^59^. Given that these two regions have different developmental origins, it suggests caution in interpretation of the role of AVPR1A in mouse and rats as it relates to adrenal function.

Some of the most robust specific binding observed in this study was localized to the uro-anogenital region. Specific AVPR1A radioligand binding was seen in the bladder, rectum, internal anogenital, and external anogenital regions. While it is known that increased AVP during birth assists in delayed voiding in newborns^5^, postnatal self-regulation of voiding is a common difficulty in altricial mammals, which can be resolved with the mother providing anogenital stimulation. Maternal anogenital licking results in the reflexive urination by the infant, allowing them to relieve themselves. Osmolarity imbalances increase the dam’s drive for NaCl and she consumes the urine produced by her offspring^60^. Due to the presence of both oxytocin and AVP that have been identified in saliva^51,52^ it may be possible that parental licking can provide an exogenous source of AVPR1A activation for neonate’s anogenital tissues. Sex specific differential treatment of pups has been documented in rats^61^, ferrets^62^, and gerbils^63^ with higher rates of anogenital licking directed toward male offspring. An olfactory basis for maternal preference for the urine of male Long-Evans rat pups has been established, adding further evidence to maternal preference for male offspring^64^. Ongoing studies within the lab aim at further assessing this question and the role of AVP and neonatal anogenital stimulation in development, and the potential for sex-specific effects.

These data show examples of specific and robust AVPR1A in neonatal mouse tissues. These findings generate hypotheses of the function of AVPR1A in the transition to post-natal life of the altricial mouse and inform the interpretations of results obtained in AVPR1A KO mouse models.

## Supplementary information

Supplementary Information.

## Data Availability

All data generated in this manuscript are included in this published article (and its Supplementary Information file).
